# Nervous Disorders from Genital Irritation

**Published:** 1876-01

**Authors:** W. M. Chamberlain


Nervous Disorders from Genital Irritation.—*By W. M. Chamberlain.*—Pertinent to the subject discussed by Dr. Jacobi, in his remarks upon nervous disorders arising from genital irritation, reported in your issue of December 4th, is the following case:

There are two reasons which dispose me to think a single case worthy of mention and record: the first is, that the subject is, as every one who has read Dr. Jacobi's remarks must feel, of great and insufficiently recognized importance in its bearing upon the development and nervous endowment of children; and the second, that the case illustrates, in a somewhat striking way, the peculiar views in regard to strictures "of extreme calibre," which Dr. Otis has the credit of having brought prominently before the profession. A gentleman, 35 years of age, a merchant, about a year since began to tell me of some trouble in his urethra. Some months before he had a clap, which was fairly cured, and left no gleet. By and by he began to suffer from a thrilling pain, which he described as radiating from a point in the urethra, half an inch behind the corona glandis, outward to the left side of the dorsum of the penis. It was not an acute pain, but it was constant. Business must be very brisk, or company very entertaining, to make him forget it, or to interrupt a sexual erethism which it excited and maintained, to his great annoyance.

He was an unmarried man, and he did not like it that he could not see a woman without feeling an improper impulse toward her.

But his great trouble was at night. The pain teased him until he got to sleep, and when consciousness was suspended he had erections so urgent and continuous that his sleep was unrefreshing and his waking languid. In short, this matter was making a change in his whole life. He had been a keen and ambitious business man, but now he did not get to his store until noon, and was becoming a flabby erotomaniac.

Examination showed some redness about the meatus, but no discharge or tenderness on pressure. Examined by bougie, he complained of sensitiveness at the spot whence the pain radiated. By the endoscope nothing was determined. Successively steel sounds, 14, 16, 18 of the English scale, were passed into the bladder without trouble. Eighteen completely filled the meatus, and even stretched it, though it passed onward without difficulty. The calibre of the passage was therefore at all points somewhat larger than at its orifice. This has been held to be conclusive evidence against the presence of stricture. Therefore at that time no larger instrument was tried. Regarding the case as a neurosis, or possibly a neuroma of the submucous tissue, various expedients were employed. The most successful were pencils of butter of cacao, containing as much iodoform as might be mixed with it. One of these was passed down to the seat of irritation, at night a full dose of bromide of potassium given, and the genitals enveloped in a cloth wetted with ice-water. He said that this programme mitigated his priapism, but obliged him to apologize to everybody for smelling so. But he kept it up for a good while, until the bromide seemed to produce a herpetic trouble of the skin. Then I suggested blistering under the track of the urethra. He approved, and wore several successive coats of cantharidal collodion, hiding his talent in a napkin for some weeks.

But all these ungracious methods appearing to be only of temporary use, I determined to stretch his urethra. With some force a bulbous bougie (31 French) was pushed through the meatus and fossa navicularis. It encountered resistance at the seat of the pain, but thence moved onward to the bulbo-membranous portion easily. In withdrawing it was arrested at the same spot, and considerable blood followed its removal. Some days after he returned to tell me that the pain and sexual irritation had been much relieved. The same process was repeatedly followed by the same result. About four days after each dilatation the irritation returned, and continued until the urethra was again stretched. This sufficiently showed that the whole train of symptoms depended upon a narrowing of the urethra, so slight as hardly to deserve the name of stricture. The case was typically an illustration of the views of Dr. Otis, who suggested, on hearing my account of it, division of the band. This I did thoroughly after freely incising the meatus, and passed bulb 39, of the French scale, down to the membranous portion. At last accounts, some six weeks after the operation, the man seemed to be entirely cured, but was passing, at intervals of a week, bulb No. 25, to prevent recontraction.

Dr. Jacobi raises the question, whether many of these cases which have been called reflex are not rather direct genital irritation, tending to an ascending neuritis, or even myelitis. Such would seem to be the solution in the present instance.—*Medical Record.*

